# Phase III Study to Confirm Clinical Similarity of MB09, a Denosumab Biosimilar, and Prolia^®^ in Postmenopausal Women with Osteoporosis (SIMBA Study) †

**DOI:** 10.3390/pharmaceutics18030291

**Published:** 2026-02-27

**Authors:** Jerzy Supronik, Elene Giorgadze, Tomasz Blicharski, Sara Sánchez-Vidaurre, Luis Pérez-Díaz, Alexandra Paravisini, Susana Millán

**Affiliations:** 1 Osteo Medic s.c. Artur Racewicz, Jerzy Supronik, 15-351 Białystok, Poland; 2 National Institute of Endocrinology, Tbilisi 0159, Georgia; 3 Department of Orthopedics and Rehabilitation, Lublin Medical University, 20-954 Lublin, Poland; 4 Medical Department, mAbxience Research S.L., 28050 Madrid, Spain

**Keywords:** biosimilar, denosumab, efficacy, MB09, pharmacology, safety

## Abstract

**Background/Objectives**: To assess the clinical similarity in terms of efficacy, pharmacodynamics (PD), pharmacokinetics (PK), safety, and immunogenicity between MB09 (denosumab biosimilar) and the reference product [RP, (Prolia^®^)] up to 18 months in women with postmenopausal osteoporosis (PMO). **Methods**: Women with PMO received three doses of 60 mg of MB09 or RP subcutaneously, every 6 months [two doses in the main treatment period and one dose in the transition period (TP)]. The primary efficacy endpoint was the percent change from baseline (%CfB) in lumbar spine bone mineral density (BMD). Secondary endpoints included other efficacy parameters and PD, PK, safety, and immunogenicity assessments. **Results**: A total of 555 subjects received MB09 (*N* = 278) or RP (*N* = 277). At month 12, %CfB in lumbar spine BMD was comparable between groups (MB09 *versus* Prolia) and met the predefined equivalence margins. Secondary efficacy endpoints—%CfB in lumbar spine BMD at 6 months and %CfB in hip and femoral neck BMD at 6 and 12 months—were similar between groups. PD marker (serum carboxy terminal cross linking telopeptide of type I collagen) was similarly suppressed in both groups, and the inhibition was maintained in the TP. PK results showed similar denosumab systemic exposure for MB09 and the RP. Both study treatments were well tolerated with similar safety profiles throughout the study period. The incidence of anti-denosumab antibodies was very low. **Conclusions**: MB09 demonstrated equivalent efficacy to the reference denosumab in women with PMO. All secondary efficacy endpoints, together with PD, PK, safety, and immunogenicity assessments, supported MB09 as a denosumab biosimilar (NCT05338086, EudraCT No. 2021-003609-24).

## 1. Introduction

Postmenopausal osteoporosis (PMO), a systemic skeletal disease caused by estrogen deficiency, leads to increased osteoclast differentiation and activation, accelerated bone resorption that outpaces formation, and rapid bone loss. This results in low bone mineral density (BMD), deteriorated bone microarchitecture, decreased bone strength, and increased risk of fragility fractures [1]. Therefore, the goal of treatment care for subjects with PMO is to prevent fractures from occurring (primary or secondary prevention) and to improve patient outcomes. Currently, PMO management includes dietary and lifestyle modifications for all types of patients; however, pharmacologic approaches are indicated only for those at high risk of fracture [2,3].

Denosumab (brand name Prolia^®^) is the first receptor activator of nuclear factor kappa-B ligand (RANKL) inhibitor that received US Food and Drug Administration (FDA) and European Medicines Agency (EMA) approval in 2010, with approved indications for the treatment of osteoporosis in postmenopausal women and in men at increased risk of fractures. Denosumab is a human monoclonal antibody (IgG2 type) that targets and binds with high affinity and specificity to RANKL, preventing activation of its receptor, RANK, on the surface of osteoclast precursors and osteoclasts. This interaction inhibits osteoclast formation, function, and survival, thereby decreasing bone resorption in cortical and trabecular bone [4].

MB09 (brand name Izamby^®^/Denbrayce^®^) is a denosumab biosimilar developed by mAbxience Research S.L. (Madrid, Spain) that was recently approved by the EMA in 2025 [5,6]. The MB09 development program was designed with the aim of demonstrating MB09 biosimilarity to the two commercially available denosumab reference products (RPs), Prolia^®^ and Xgeva^®^ [Amgen Inc. (Thousand Oaks, CA, USA)] [4,7]. Consequently, a stepwise approach was followed for its development. Once analytical similarity was demonstrated *in vitro*, a first clinical study aiming to establish the pharmacokinetic (PK) bioequivalence was conducted in healthy volunteers using Xgeva^®^ as the RP [8]. This study demonstrated similar PK profiles between MB09 and the RP and provided strong evidence for the biosimilarity of MB09 to reference denosumab. Subsequently, to complete the totality of evidence and to further support the similarity between MB09 and the RP, the last step in the MB09 clinical development program included one phase III confirmatory clinical study designed to compare the efficacy, pharmacodynamics (PD), PK, safety, and immunogenicity profiles between MB09 and the RP (Prolia^®^) in women with PMO for a total duration of 18 months, including the assessment of a single switch from the RP to MB09 (SIMBA study). Preliminary results from this phase III study were previously presented as a conference abstract [9]. The current study presents the main findings from the completed confirmatory phase III study to demonstrate therapeutic similarity between MB09 and the RP, focusing on efficacy and safety data over the total study duration.

## 2. Materials and Methods

### 2.1. Study Design

**Figure 1:**
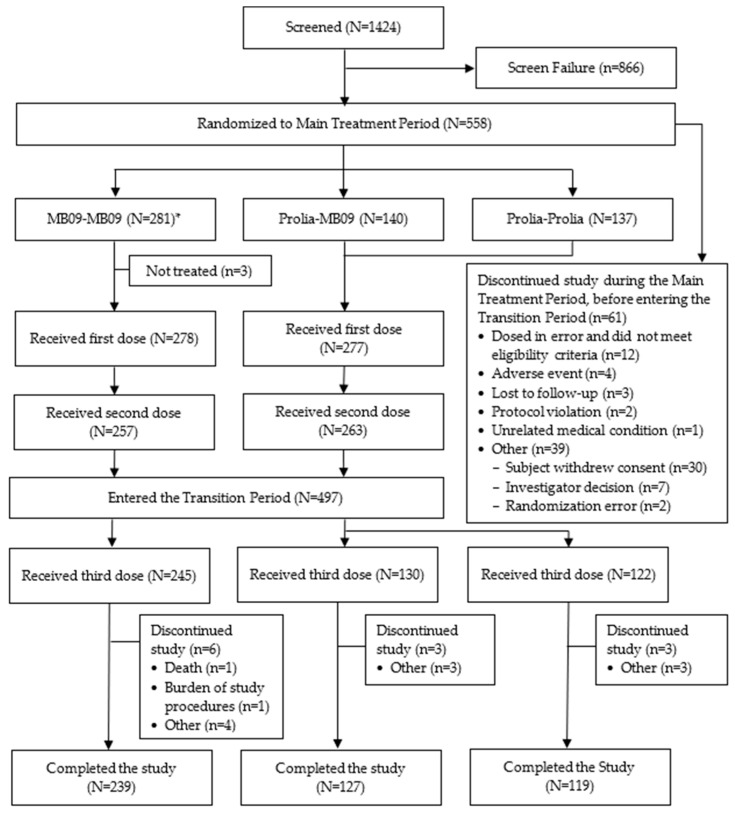
Subjects disposition. * *N* as randomized. One subject randomly assigned to the MB09-MB09 arm did not receive the assigned treatment and instead received study treatment assigned to the RP-RP arm throughout the study. This subject was analyzed for efficacy per the treatment as randomized (MB09-MB09) and for safety per the actual treatment received (RP-RP).

SIMBA was a randomized, double-blind, three-parallel arms, multicenter, and multinational phase III study conducted in 56 sites, including the following eight countries: Bulgaria, Estonia, Georgia, Hungary, Latvia, Poland, Serbia, and Mexico. The study was registered in clinicaltrials.gov (NCT05338086) and EudraCT (Number: 2021-003609-24) and approved by the Ethics Committee (EC) at each site before implementation. The study was conducted in accordance with the ethical principles of the Declaration of Helsinki, International Conference on Harmonisation (ICH) E6 Guideline for Good Clinical Practice, and Council for International Organizations of Medical Sciences Ethical Guidelines, and all participants provided written informed consent prior to their participation in the study. The study is reported in accordance with the Consolidated Standards of Reporting Trials (CONSORT) 2025 and the diagram for documenting the flow of participants through the study is provided in Figure 1.

Eligible subjects were postmenopausal women with osteoporosis aged ≥55 and ≤80 years, with body weight ≥50 and ≤99.9 kg, body mass index (BMI) of ≤30 kg/m^2^, absolute BMD consistent with T-score −4.0 and −2.5 at the lumbar spine or total hip as measured by dual-energy X-ray absorptiometry (DXA) during the screening period, with at least two intact, nonfractured vertebrae in the L1 to L4 region and at least one hip joint evaluable by DXA. Key exclusion criteria included the following: previous exposure to denosumab; any other investigational agent for osteoporosis; intravenous bisphosphonate, strontium, or fluoride for osteoporosis within 5 years of screening; oral bisphosphonates with ≥12 months cumulative use; or subjects with ongoing use of any osteoporosis treatment taken within 5 years prior to screening and other bone-active agents.

The SIMBA study was designed to investigate the efficacy, PD, PK, safety, and immu-nogenicity profiles of MB09 compared to Prolia sourced from Europe (EU-Prolia). The study comprised two periods: a main treatment period (MTP) (from day 1 to month 12), where two doses of the study treatment were administered, and a transition period (TP) (from month 12 to month 18), where one dose of the study treatment was administered. In the MTP, subjects were randomly assigned in a 2:1:1 (MB09 *versus* RP *versus* RP) ratio by interactive response technology to one of the three arms of the study: in Arm 1 MB09-MB09, subjects were to receive two doses of MB09 at day 1 and at month 6; in Arm 2 Prolia-MB09, subjects were to receive two doses of the RP at day 1 and at month 6; and in Arm 3 Prolia-Prolia, subjects were to receive two doses of the RP at day 1 and at month 6. In the TP, subjects assigned to Arm 1 and Arm 2 received one dose of MB09 at month 12, and subjects assigned to Arm 3 received one dose of the RP at month 12, so the TP included 2 arms, where one of the treatment arms switched from one of the RP’s arms to the MB09 arm, being the ratio of subjects who received MB09 *versus* RP 2:1 (following the scheme: MB09-MB09; Prolia-MB09; Prolia-Prolia).

The randomization was stratified by baseline BMD T-score at the lumbar spine (≤−3.0 and >3.0), BMI (<25 kg/m^2^ and ≥25 kg/m^2^), age at study entry (≥55 to <68 years and ≥68 to ≤80 years), and prior bisphosphonate medication use at study entry (prior use of bisphosphonates and no prior bisphosphonate use).

MB09 or RP were administered as a subcutaneous (SC) injection (60 mg) every 6 months, for a total of three doses, until month 12, where a last dose was administered. All subjects received at least 1000 mg of elemental calcium and at least 400 IU vitamin D daily from randomization until the end of the study. These doses could be adjusted based on the investigators’ criteria.

The EU-Prolia used as the comparator in this study was obtained through the authorized European supply chain, and all batches administered were released from Germany (batch numbers: 1136812, 1142161, and 1153835).

### 2.2. Assessments

BMD of the lumbar spine, total hip, and femoral neck were conducted using DXA and assessed by an independent radiology review committee at months 6 and 12 of the study. Lateral spine radiographs were performed at screening, and any additional radiographs were performed if required during the study for new clinical fractures confirmation.

For PD analysis, serum carboxy-terminal cross-linking telopeptide of type I collagen (sCTX) was measured in fasting samples at the following timepoints: day 1 (0 pre-dose), day 11, day 36, day 90, and day 182 (pre-dose), during the first and the third dose of study treatment. Serum samples were analyzed at the central laboratory using a validated enzyme-linked immunosorbent assay method (quantification range: 70.0–1130 pg/mL) (further details available in the Supplementary Information Methods).

For PK analysis, denosumab serum concentrations were collected at the following timepoints: day 1 (0 pre-dose), day 11, day 36, day 90, and day 182 (pre-dose), during the first and the third dose of study treatment. PK samples were analyzed at the central laboratory using a validated MesoScale Discovery-based electrochemiluminescence method (quantification range: 20.0–800 ng denosumab/mL) (further details available in the Supplementary Information Methods).

Safety assessments were performed throughout the study and included the report of the treatment-emergent adverse events (TEAEs), serious adverse events (SAEs), and adverse events of special interest (AESIs) [injection site reaction, drug-related hypersensitivity/allergic reaction, infection, hypocalcemia, osteonecrosis of the jaw (ONJ), dermatologic reaction, and atypical femoral fracture]. TEAEs were coded according to the Medical Dictionary for Regulatory Activities (MedDRA; version 24.1) and graded based on the US National Cancer Institute Common Terminology Criteria for Adverse Events (NCI-CTCAE, version 5.0). Other safety assessments included the following: physical examination and vital signs and body weight measurements; lateral spine radiography (at screening only and performed after randomization if required by the investigator); electrocardiogram (ECG) and clinical laboratory analyses (hematology, clinical chemistry, coagulation, and urinalysis); and the recording of prior and concomitant medications.

The immunogenicity of denosumab was determined by detection of anti-drug antibodies (ADAs) in serum samples at the following timepoints: day 1 (0 pre-dose), day 11, day 36, day 90, and day 182 (pre-dose), during the first and the third dose of the study treatment. ADAs incidence, titers, and their neutralizing (Nabs) activity were assessed at the central laboratory using a validated immunoassay (further details available in the Supplementary Information Methods). Treatment-induced ADAs (TI-ADAs) during the TP were defined as an ADA-negative status at month 12 followed by any ADA-positive result during months 12–18. Treatment-boosted ADAs (TB-ADAs) were defined as ≥4-fold titer increases from pre-switch to any post-switch assessment. Nabs were assessed in ADA-positive samples.

A data and safety monitoring board (DSMB) reviewed safety results and the benefit-risk assessment of the treatment received periodically on an ongoing basis during the study.

### 2.3. Study Endpoints

The primary efficacy endpoint was the percent change from baseline (%CfB) in lumbar spine BMD after 52 weeks of treatment. The secondary efficacy endpoints included the evaluations of %CfB in lumbar spine BMD after 6 months, hip BMD after 6 and 12 months, and femur neck BMD after 6 and 12 months.

Other secondary endpoints included the following assessments: PD profile, evaluated by means of concentration of sCTX and %CfB (%inhibition) values of sCTX, area under the effect curve (AUEC) and area under the percent inhibition curve (AUIC), respectively, up to month 6 in the MTP and in the TP; PK profile, in terms of maximum observed serum concentration after study treatment administration (C_max_) in both the MTP and the TP, area under the concentration-time curve (AUC) over the first 6-month dosing interval (AUC_0–6 months_) in the MTP and in the TP, and minimum concentration (C_trough_) at months 6 and 12 in the MTP and at month 6 in the TP. Safety and immunogenicity assessments were also evaluated as secondary endpoints in both study periods.

### 2.4. Statistical Methods

To ascertain the therapeutic equivalence between MB09 and the RP, a mixed model for repeated measures (MMRM) fitted to the composite %CfB lumbar spine BMD was used. The MMRM included terms for visit by treatment, with stratification variables (age, BMI, and prior use of bisphosphonates) included as classification factors and baseline BMD included as a continuous covariate. The subject was included as a random effect. The estimated mean difference in %CfB lumbar spine BMD at month 12 was presented with 95% confidence interval (CI) and equivalence was concluded if this fell within the predefined equivalence margins of [−1.45%, 1.45%] based on the modified full analysis set (mFAS). The ±1.45% equivalence margins was derived from a meta-analysis of three denosumab clinical trials [10,11,12], preserving ≥70% of the lower 95% CI bound of the pooled lumbar spine BMD effect at 12 months. Using a standard deviation of 4.5%, and assuming 15% of dropout, a total sample size of 528 subjects was required to provide 85% power for the demonstration of equivalence at α = 2.5%.

mFAS included all eligible treated subjects but excluded data observed after the first occurrence of those intercurrent events where a hypothetical strategy was taken (such as missing a dose, errors or deviations in dosing, or receipt of any prohibited therapies or other osteoporosis medications). In addition, sensitivity analyses were conducted to explore assumptions around missing data and data expected in the hypothetical scenario that both doses had been taken by all subjects.

Following the primary efficacy endpoint approach, an MMRM was used to analyze the composite endpoint of %CfB on the mFAS in the following: lumbar spine BMD after 6 months; hip BMD after 6 and 12 months; and femur neck BMD after 6 and 12 months (no equivalence margins were set for secondary efficacy endpoints).

To compare the denosumab PD profile between MB09 and the RP, PD parameters such as AUEC from time zero to 6 months (AUEC_0–6 months_), and AUIC from time zero to 6 months (AUIC_0–6 months_) were generated. For the PD statistical analysis, AUEC_0–6 months_ and AUIC_0–6 months_ were analyzed on the log scale by analysis of covariance (ANCOVA). The ratios (MB09 compared to RP) of the geometric least square means (GLSMs) and the corresponding 90% CIs for the ratios were computed. Biosimilarity with respect to PD was concluded if the 90% CI for MB09 to the RP ratios of the GLSMs were entirely contained within the [80.00%, 125.00%] interval for AUEC and AUIC.

To compare denosumab PK profiles between MB09 and the RP, the following PK parameters were generated by the noncompartmental analysis for each individual subject: C_max_, AUC_0–6 months_, and C_trough_. For PK statistical analysis, C_max_ and AUC_0–6 months_ were analyzed on the log scale by ANCOVA. The model included treatment and stratification variables as fixed effects. The estimated mean differences with 90% were back transformed to give the ratio of GLSMs (MB09/RP) with 90% CIs following the first treatment in the MTP. Similarly, parameters derived in the TP were analyzed and comparisons were made (Arm 2 Prolia-MB09/Arm 3 Prolia-Prolia and Arm 1 MB09 MB09/Arm 3 Prolia-Prolia).

Safety and immunogenicity analyses were performed on the safety analysis set, consisting of all randomized subjects who received at least one dose of MB09 or RP, and results were compared between treatment groups in both study periods.

All statistical analyses were performed using Statistical Analysis System (SAS^®^) software version 9.4 (SAS Institute Inc., Cary, NC, USA). PD and PK parameters were generated for each individual subject, if data permitted, by the noncompartmental analysis using Phoenix WinNonlin software Version 8.3 (Certara USA, Inc., Princeton, NJ, USA).

## 3. Results

### 3.1. Subjects Disposition and Demographics

Between March 2022 and December 2023, a total of 1424 subjects were screened, 558 of which were randomized to receive MB09 or the RP (281 and 277, respectively). Three subjects out of the randomized 558 did not receive study treatment; thus, a total of 555 subjects were treated (MB09 or RP) at the first dose. The second dose of the study treatment was administered in 520 (93.7%) of the initially treated subjects. A total of 61 subjects (10.9%) discontinued the study during the MTP: 36 subjects (12.8%) in the MB09 group and 25 subjects (9.0%) in the RP group (Figure 1).

At month 12, 497 subjects entered the TP to receive the third dose of the study treatment: 245 subjects in the MB09-MB09 arm; 130 subjects in the RP-MB09 arm; and 122 subjects in the RP-RP arm. Of those, 12 subjects (2.4%) discontinued the study: 6 subjects (2.4%) in the MB09-MB09 arm, 3 subjects (2.3%) in the RP-MB09 arm, and 3 subjects (2.5%) in the RP-RP arm. Reasons for discontinuation from the study were balanced between treatment arms in both the MTP and the TP, with withdrawal of informed consent being the most common reason for treatment discontinuation in both study periods (Figure 1).

Demographic characteristics of study subjects were well balanced across treatment groups (Table 1). Overall, mean age was 65.8 years, where 38.4% of the subjects (*n* = 213) were older than 68 years; mean weight was 63.2 kg with a mean BMI of 24.7 kg/m^2^, where >40% of the subjects showed BMI ≥ 25 kg/m^2^. Most subjects were White by race (>99.3%) and not Hispanic or Latino by ethnicity (>95.9%). Other important baseline characteristics, such as smoking status, were also balanced between both treatment groups, where more than 60% of the recruited subjects never smoked.

Baseline disease characteristics were also well balanced across treatment groups, with a mean serum calcium of 2.4 mmol/L, mean serum creatinine of 58.9 µmol/L, mean serum thyroid stimulating hormone (TSH) of 1.89 mIU/L, mean serum parathyroid hormone (PTH) of 39.3 ng/L, and mean serum 25-OH-vitamin D of 84.9 nmol/L. The mean lumbar spine BMD was 0.770 g/cm^2^, with a total of 51.7% and 48.3% of subjects having the baseline BMD T-score at the lumbar spine >−3.0 and ≤−3.0, respectively. The mean total hip and femur neck BMD were around 0.7 g/cm^2^. A total of 210 subjects (37.8%) had a history of fractures (any fracture); of these, 68 subjects (32.4%) had vertebral fractures (Table 1).

### 3.2. Efficacy

Of the 555 subjects who received at least one dose of either MB09 (*N* = 277 subjects) or the RP (*N* = 278 subjects), a total of 515 subjects (92.3%) completed at least one lumbar spine, hip, or femur neck BMD assessment after one year of treatment.

For the primary efficacy endpoint, %CfB in lumbar spine BMD, results demonstrated that MB09 was therapeutically equivalent to the RP, since the difference in LS means (95% CI) between both products was 0.20 (−0.51, 0.91), which fell entirely within the predefined margins of [−1.45, 1.45]. Further sensitivity analyses, which explored assumptions around missing data and data expected in the hypothetical scenario that both doses had been taken by all subjects, were conducted, and similar results to the primary MMRM without any imputation were obtained. Briefly, the difference in LS means (95% CI) in %CfB in lumbar spine BMD between MB09 and the RP after sensitivity estimation using MMRM after multiple imputation was 0.20 (−0.51, 0.90), falling into the predefined margins of [−1.45, 1.45] and supporting the primary efficacy analysis.

Similarly, all secondary efficacy endpoints —%CfB in lumbar spine BMD at month 6, %CfB in hip BMD at month 6 and month 12, and %CfB in femur neck BMD at month 6 and month 12—were consistent with the primary efficacy endpoint analysis. The difference in LS means (95% CI) were as follows: 0.07 (−0.55, 0.69) at the lumbar spine at month 6; −0.17 (−0.61, 0.27) and 0.10 (−0.39, 0.59) at the hip at month 6 and month 12, respectively; 0.25 (−0.35, 0.86) and 0.36 (−0.28, 1.00) at the femur neck at month 6 and month 12, respectively (Table 2).

Throughout the study, fracture events were uncommon; a total of 22 fractures were reported in 22 subjects. In the MTP, a small number of fractures occurred in each treatment group (10 fractures in the MB09 group and 6 fractures in the RP group), and only three fractures were classified as non-traumatic based on the investigator’s assessment. During the TP, fracture incidence remained minimal, with a total of six fractures reported, divided into two fractures in each treatment arm (MB09-MB09 arm, RP-MB09 arm, RP-RP arm). Because fractures were neither predefined as a secondary nor exploratory efficacy endpoint and the number of events was very low, these findings are presented descriptively to avoid overinterpretation.

### 3.3. PD

**Figure 2:**
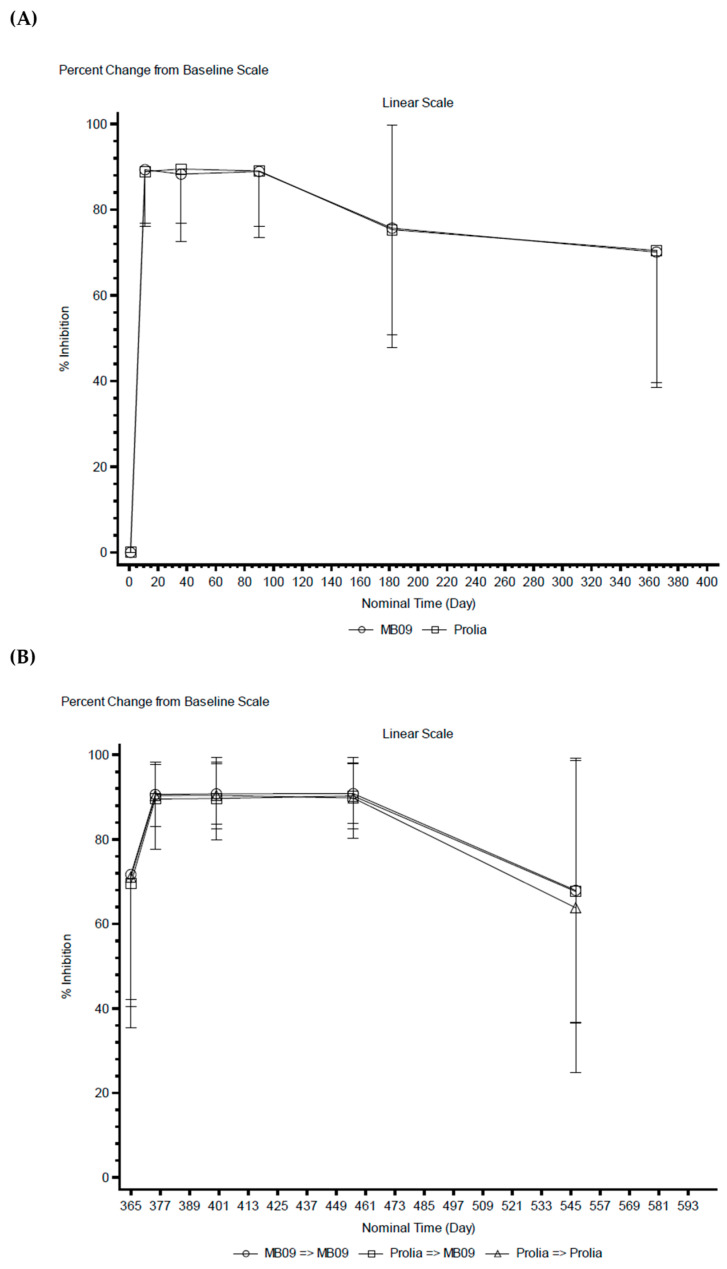
(**A**) Mean (±SD) %CfB sCTX concentrations *versus* time following subcutaneous administration—MTP; (**B**) Mean (± SD) %CfB sCTX concentrations *versus* time following subcutaneous administration—TP. Abbreviations: %CfB, percent change from baseline; MTP, main treatment period; sCTX, serum carboxy-terminal cross-linking telopeptide of type I collagen; SD, standard deviation; TP, transition period.

Baseline sCTX levels (day 1 and month 12) were comparable across treatment groups [arithmetic mean (% of coefficient of variation): 556 pg/mL (57.4%) for MB09 and 529 pg/mL (55.1%) for RP at day 1, and 133 pg/mL (103%) for the MB09-MB09 arm, 130 pg/mL (88.2%) for the RP-MB09 arm, and 131 pg/mL (113%) for the RP-RP arm]. After the first two denosumab administrations, sCTX concentrations decreased rapidly, with mean %CfB values (representing mean %inhibition) of 75.7% at month 6 and 70.1% at month 12 in the MB09 group, and 75.3% at month 6 and 70.5% at month 12 in the RP group (Figure 2A). In the TP, profiles of sCTX and %CfB (%inhibition) of sCTX were comparable across all treatment arms, as concentrations decreased rapidly and remained suppressed through TP month 3 and trended to return to TP baseline (month 12) levels at month 6 (Figure 2B).

Statistical analysis of PD parameters of sCTX in the MTP showed similar behavior between both groups. In terms of absolute sCTX levels (AUEC_0–6 months_), both groups were comparable, with a GLSM ratio (90% CI; MB09/RP) of 99.91% (93.22, 107.08). Similar results were obtained for %CfB in sCTX levels (AUIC_0–6 months_), where the GLSM ratio (90% CI; MB09/RP) was 99.13% (96.76, 101.55). For both PD parameters, the GLSM ratios were within the predefined acceptance limits, demonstrating equivalence of MB09 to the RP (Table 3).

In the TP, geometric means AUEC_0–6 months_ in sCTX were similar across the three treatment arms; 10,700 day·pg/mL for the MB09-MB09 arm, 11,000 day·pg/mL for the RP-MB09 arm, and 9770 day·pg/mL for the RP-RP arm. In terms of geometric mean %CfB AUIC_0–6 months_ in sCTX, the MB09-MB09 and RP-MB09 arms presented a value of 15,500 day·%, very similar to the value of 15,600 day·% observed for the RP-RP arm.

### 3.4. PK

Denosumab systemic exposure, in terms of C_max_, AUC_0–6 months_, and C_trough_, was equivalent between treatment groups, as the 90% and 95% CIs around the GLSM ratios fell within the predefined equivalence limits in all instances in both the MTP and the TP, respectively, demonstrating equivalence of MB09 to the RP. Specifically, in the MTP, the GLSM ratios between MB09/RP (90% CI) for C_max_, AUC_0–6 months_, month 6 C_trough_, and month 12 C_trough_ were 104.13% (99.66%, 108.79%), 106.06% (100.82%, 111.57%), 103.10% (89.47%, 118.81%), and 103.25% (86.84%, 122.75%), respectively. In the TP, the GLSM ratios between MB09-MB09 and RP-RP (95% CI) for C_max_, AUC_0–6 months_, and month 6 C_trough_ were 103.40% (96.28%, 111.04%), 101.57% (93.68%, 110.12%), and 91.60% (68.36%, 122.75%), respectively, and for the ratio RP-MB09 to RP-RP for C_max_, AUC_0–6 months_, and month 6 C_trough_ were 103.65% (95.66%, 112.31%), 99.48% (90.80%, 108.99%), and 86.17% (61.93%, 119.90%), respectively (Table 3).

### 3.5. Safety

During the MTP, the incidence of TEAEs was comparable across treatment groups: 58.1% in the MB09 group and 54% in the RP group (Table 4). Most of these TEAEs were mild or moderate in severity, with no remarkable differences between treatment groups at each severity level. The most frequently reported TEAEs were upper respiratory tract infection, nasopharyngitis, arthralgia and COVID-19 infection. No differences were observed when comparing both groups (Supplementary Table S1). The treatment-related TEAEs were higher in incidence in the MB09 group than in the RP group [41 (14.8%) in the MB09 group *versus* 24 (8.6%) in the RP group]. The most common related events were increased blood PTH, urinary tract infections, decreased blood calcium, and pain-related events.

A total of 37 serious TEAEs were experienced by 19 (6.9%) and 13 (4.7%) subjects in the MB09 and the RP groups, respectively. The most common serious events were those related to fractures, followed by gastrointestinal disorders and hepatobiliary disorders, with no clear trend between treatment groups, and only two were considered related to the study treatment (an event of ONJ in the MB09 group and a migraine event in the RP group). AESIs were reported by similar proportions of subjects across groups: 28.9% in the MB09 group and 27% in the RP group, with the most common reported AESIs being classified as infections and infestations, dermatologic reactions, hypocalcemia, and injection site reactions. Serious AESIs were reported by only four subjects (1.4%) in the MB09 group: an event of pulmonary tuberculosis in one subject, pneumonia in one subject, psoriasis in one subject, and an event of ONJ in one subject.

Throughout the study, the overall incidence of TEAEs was similarly distributed across the treatment arms, as displayed by treatment received during the MTP and in the TP (kept MB09, switched from RP to MB09, or kept RP) (Table 4). Specifically, for the TP analysis, TEAEs reported in subjects who switched from RP to MB09 were compared with the safety outcomes of subjects who continued on RP (RP-RP) during the same period. The most frequently reported TEAEs were consistent with those observed during the MTP, were generally mild to moderate in severity, and were mostly expected for this age group (Supplementary Table S2). TEAEs related to the study treatment were similar in frequency between treatment groups: three subjects (2.3%) in the RP-MB09 group and five (4.1%) in the RP-RP group).

Serious TEAEs of Grade 2 (diverticulitis and thrombophlebitis) and 3 (cardiac disorder) were reported by two subjects (one subject each) in the RP-RP group, but none was considered related to the study treatment. A total of 45 AESIs were reported by 38 subjects, with similar incidence in the RP-MB09 and RP-RP groups: 17 (13.1%) subjects with 21 events and 21 (17.1%) subjects with 24 events, respectively; these events were considered not serious in nature and were most commonly reported as infections and infestations and dermatologic reactions. One subject receiving MB09 throughout the study (MB09-MB09) experienced a TEAE of pneumonia that led to the subject’s death in the TP; the event was considered unrelated to the study treatment.

### 3.6. Immunogenicity

During the MTP, TI-ADAs were infrequent, applying to six subjects (1.1%), all of whom receiving the RP treatment. All ADAs detected during this period showed no neutralizing activity. During the TP, a detailed immunogenicity evaluation was performed to specifically evaluate ADA emergence after switching from RP to MB09. Importantly, no TI-ADAs occurred in the switch arm (RP-MB09) nor in the maintenance RP arm (RP-RP). The only TI-ADAs detected during this period occurred in a subject (0.4%) who received MB09 in both study periods (MB09-MB09). All ADA positive cases observed in the RP-MB09 and RP-RP arms during the TP corresponded to subjects who presented an ADA positive response at month 12 and, therefore, did not represent treatment-induced events but a persistent ADA response. No neutralizing activity was detected in any treatment arm.

## 4. Discussion

This phase III confirmatory study demonstrated similar efficacy, PD, PK, safety, and immunogenicity of denosumab biosimilar MB09 and the reference denosumab in postmenopausal women with osteoporosis over 18 months. The primary efficacy endpoint, mean %CfB in lumbar spine BMD at 12 months, demonstrated similar clinical efficacy between MB09 and the RP as the LS mean treatment difference (95% CI) was 0.20 (−0.51, 0.91), which was entirely contained within the predefined equivalence margins [−1.45, 1.45]. Moreover, the sensitivity analysis using MMRM (after MI for the missing lumbar spine BMD values at 12 months) provided additional evidence for similar efficacy between MB09 and the RP and supports the robustness of the primary analysis. These findings were further supported by the analysis of secondary efficacy endpoints lumbar spine BMD at 6 months and hip and femur neck BMD at months 6 and 12, where treatment comparisons (95% CIs) were within −1 to 1, reinforcing the claim for MB09 biosimilarity.

The efficacy results after 12 months of treatment obtained from this study (i.e., the around 5% increase in BMD at the lumbar spine) are in line with historical data from the RP [11] and with data from other denosumab biosimilar studies [13,14,15,16]. More importantly, an improvement in lumbar spine BMD of this magnitude corresponds to a clinically significant difference in vertebral fracture rate [10,11]. In fact, it has been reported that a 2% increase in lumbar spine BMD is associated with a 28% reduction in vertebral fracture [17]. In this study, fractures rates were low (˂4%) in both MB09 and RP groups throughout the study period, in line with low levels reported from the RP [11] and other denosumab biosimilar products [14]. In addition vertebral fractures were only reported by one patient receiving MB09 in the MTP and by two patients (one in each treatment group) in the TP. These findings demonstrate the therapeutic similarity between MB09 and the RP in terms of increasing BMD and reducing the fractures rates in this population.

Besides the BMD results, PD equivalence was assessed in this study by the sCTX biomarker, an indicator of bone resorption. An equivalent inhibition of sCTX levels was observed after the administration of either MB09 or the RP. sCTX concentrations decreased rapidly and largely and remained suppressed until the end of the 18-month treatment period, which demonstrates the efficacy of both treatments in inhibiting bone resorption. Comparable results were also observed in the TP across the three treatment arms, which confirms there was no impact from switching from RP to MB09 on bone metabolism. These results are aligned with sCTX results from similar switching studies, demonstrating the maintenance of sCTX levels suppression after switching from RP to the biosimilar product [13,14,15]. As demonstrated by the statistical analysis, the 90% CIs for both the absolute sCTX levels (AUEC_0–6 months_) and %CfB values in sCTX levels (AUIC_0–6 months_) in the MTP and the TP have been demonstrated to be sufficiently narrow and close enough to 1 to confirm the PD similarity between MB09 and the RP. These PD findings, together with the efficacy data obtained at 12 months, support and foresee an equivalent therapeutic performance of MB09 with respect to reference denosumab.

The present study also compared the PK profiles of MB09 and the RP. In terms of C_trough_, the variability, as expected, was high for 6 months and 12 months across treatment groups, although the GLSM ratios at both timepoints fell within the equivalence margins. For C_max_ and AUC_0–6 months_ in both the MTP and the TP, results demonstrated that the ratios (MB09/RP) of the GLSM were fully contained within the equivalence margins. These PK results support the PK similarity of MB09 and the RP in the target population.

MB09 showed a similar safety profile to the RP and was well tolerated in this study. No clinically meaningful differences were noted in terms of frequency, severity, distribution, and type of TEAEs between both treatment groups in the MTP nor after switching from the RP to MB09; globally, the safety findings were consistent with the known safety profile of reference denosumab [4,7,10]. There was a slight difference in the treatment-related TEAEs distribution in favor of the biosimilar group. This imbalance was mainly driven by a higher incidence in the report of increased PTH and pain-related adverse events. None of these events were considered clinically relevant, as PTH increase is an expected physiological response after treatment with denosumab, and the events related to pain are expected events very commonly occurring after denosumab treatment. No new safety concerns were identified in this study.

With regards to immunogenicity, the results obtained in the present study show denosumab as a molecule with a low immunogenic potential. The very low ADAs incidence observed in both the MTP and the TP, with no positive Nabs results, is in line with the reported data for the RP, where the incidence of anti-denosumab antibodies stands below 1% [4,7]. Importantly, a detailed analysis during the TP demonstrated that no TI-ADAs emerged after switching from the RP to MB09, nor in subjects who continued on RP. The only ADA-positive response identified in the TP occurred in the MB09-MB09 arm (1 subject, 0.4%), and all other ADA-positive results reflected pre-existing ADAs status at month 12. No Nabs were detected, and no ADAs-related safety, PK, or PD impact was observed. These data confirm that switching from the RP to MB09 does not increase the immunogenicity risk, addressing a key biosimilarity concern.

Due to the low incidence of ADAs, no meaningful assessment could be performed to evaluate the impact of TI-ADAs on the efficacy assessment, although their occurrence did not appear to impact the efficacy results or the safety profile of denosumab in this study.

The main limitation of this study is related to the way in which the efficacy assessment was designed in the present study compared to previous studies with the RP, as the efficacy assessment was performed by means of a surrogate instead of a clinical endpoint. In the development of new osteoporosis treatments, efficacy endpoints, such as the demonstration of a decrease in the incidence of fractures, have been considered the gold standard; however, these type of clinical studies with a design focused on the efficacy evaluation of potential treatments on fracture risk are substantial, long in duration, and expensive [18]. In the case of biosimilar development, as with MB09, the clinical confirmatory study does not aim to demonstrate additional efficacy *per se*, since this has already been established for the RP [19]. Thus, in such a biosimilar confirmatory study, BMD offers a validated efficacy endpoint to detect clinically meaningful differences, if they exist, between the biosimilar and the RP. Hence, the present study does not allow for a proper comparison of the incidence of fracture events between the two treatment groups after one year of treatment but rather a descriptive analysis of the fracture events. Therefore, to strengthen the clinical relevance of the efficacy results obtained, a very sensitive PD marker of bone resorption, sCTX, was included in the study design. sCTX was able to show significant and dynamic changes after treatment administrations, and demonstrated PD equivalence between MB09 and the reference denosumab, providing further evidence to support the MB09 biosimilarity.

## 5. Conclusions

In this confirmatory phase III study in women with PMO, MB09 demonstrated a similar therapeutic performance compared to the reference denosumab in terms of efficacy, measured as changes in BMD and PD effects after 12 months of treatment. Comparable results were also obtained for PK, safety, and immunogenicity between treatment groups. Likewise, similarity was maintained up to 18 months, with no impact from switching from the RP to MB09 compared to continued RP treatment. This study supports the clinical equivalence of MB09 in postmenopausal women with osteoporosis and confirms its biosimilarity to reference denosumab. Within the totality-of-evidence framework, and considering the comprehensive analytical, non-clinical, and clinical comparability data generated for MB09, the findings from this study are consistent with the level of similarity expected for biosimilar denosumab products.

## Figures and Tables

**Table 1:** Summary of subject demographics and disease characteristics at baseline.

**Demographics at baseline**	**MB09 (*N* = 277)**	**Prolia (*N* = 278)**	**Total (*N* = 555)**
**Age (years)**			
Mean (SD)	65.8 (6.00)	65.9 (5.90)	65.8 (5.94)
**Age group, *n* (%)**			
≥55 to <68 years	170 (61.4)	172 (61.9)	342 (61.6)
≥68 to ≤80 years	107 (38.6)	106 (38.1)	213 (38.4)
**Smoking status, *n* (%)**			
Current Smoker	67 (24.2)	65 (23.4)	132 (23.8)
Former Smoker	39 (14.1)	35 (12.6)	74 (13.3)
Never-Smoker	171 (61.7)	178 (64.0)	349 (62.9)
**Race, *n* (%)**			
White	276 (99.6)	275 (98.9)	551 (99.3)
American Indian or Alaska Native	1 (0.4)	3 (1.1)	4 (0.7)
**Ethnicity, *n* (%)**			
Hispanic or Latino	10 (3.6)	13 (4.7)	23 (4.1)
Not Hispanic or Latino	267 (96.4)	265 (95.3)	532 (95.9)
**Baseline height (cm)**			
Mean (SD)	159.97 (6.252)	159.99 (6.131)	159.98 (6.186)
**Baseline weight (kg)**			
Mean (SD)	63.063 (8.8299)	63.328 (8.7580)	63.196 (8.7870)
**Baseline BMI (CRF) (kg/m^2^) ^1^**			
Mean (SD)	24.629 (3.0184)	24.737 (3.0661)	24.683 (3.0401)
**Baseline BMI group, *n* (%)**			
≥25 kg/m^2^	115 (41.5)	122 (43.9)	237 (42.7)
<25 kg/m^2^	162 (58.5)	156 (56.1)	318 (57.3)
**Disease characteristics at baseline**			
**Menopause duration (years)**			
Mean (SD)	16.422 (7.0597)	16.991 (7.3539)	16.707 (7.2074)
**Osteoporosis duration (years)**			
Mean (SD)	2.656 (4.3900)	2.042 (3.8967)	2.348 (4.1579)
**Prior use of bisphosphonates, *n* (%)**			
Yes	25 (9.0)	21 (7.6)	46 (8.3)
No	252 (91.0)	257 (92.4)	509 (91.7)
**Fracture history, *n* (%)**			
Yes	108 (39.0)	102 (36.7)	210 (37.8)
No	169 (61.0)	176 (63.3)	345 (62.2)
**History of vertebrae** **fractures, *n* (%) ^1^**			
Yes	37 (34.3)	31 (30.4)	68 (32.4)
No	71 (65.7)	71 (69.6)	142 (67.6)
**Serum calcium (mmol/L)**			
Mean (SD)	2.317 (0.083)	2.321 (0.088)	2.319 (0.085)
**Serum creatinine (µmol/L)**			
Mean (SD)	59.9 (12.66)	58.0 (12.05)	58.9 (12.38)
**Serum TSH (mIU/L)**			
Mean (SD)	1.9063 (1.29)	1.8720 (1.58)	1.8891 (1.44)
**Serum PTH (ng/L)**			
Mean (SD)	40.5 (14.64)	38.0 (12.42)	39.3 (13.62)
**Serum 25-OH-Vitamin D (nmol/L)**			
Mean (SD)	83.8 (21.50)	85.9 (26.55)	84.9 (24.16)
**Baseline BMD T-score** **at the lumbar spine (SD), *n* (%)**			
>−3.0	144 (52.0)	143 (51.4)	287 (51.7)
≤−3.0	133 (48.0)	135 (48.6)	268 (48.3)
**Lumbar spine BMD (g/cm^2^)**			
Mean (SD)	0.766 (0.0878)	0.773 (0.0862)	0.770 (0.0870)
**Total hip BMD (g/cm^2^)**			
Mean (SD)	0.731 (0.0973)	0.745 (0.0946)	0.738 (0.0961)
**Femur neck BMD (g/cm^2^)**			
Mean (SD)	0.672 (0.1085)	0.685 (0.1084)	0.679 (0.1086)
**sCTX (pg/mL)**			
Mean (SD)	556 (319)	529 (291)	N/A ^2^

Abbreviations: BMD, bone mineral density; BMI, body mass index; CRF, case report form; N/A, not available; PTH, parathyroid hormone; SD, standard deviation; sCTX, serum carboxy-terminal cross-linking telopeptide of type I collagen; TSH, thyroid stimulating hormone. ^1^ Percentages were calculated out of those who had a fracture. ^2^ sCTX concentrations were analyzed by treatment group, and values corresponding to total study population were not calculated.

**Table 2:** Means in %CfB in lumbar spine, hip, and femur neck at months 6 and 12.

%CfB in BMD ^1^	MB09 (*N* = 258)	Prolia (*N* = 266)
**Lumbar spine at month 12**		
LS mean %CfB (*n*)	5.86% (233)	5.66% (250)
**Lumbar spine at month 6**		
LS mean %CfB (*n*)	4.03% (246)	3.96% (258)
**Hip at month 12**		
LS mean %CfB (*n*)	3.37% (232)	3.28% (252)
**Hip at month 6**		
LS mean %CfB (*n*)	2.29% (241)	2.46% (257)
**Femur neck at month 12**		
LS mean %CfB (*n*)	2.75% (232)	2.39% (252)
**Femur neck at month 6**		
LS mean %CfB (*n*)	2.18% (241)	1.93% (257)

Abbreviations: BMD, bone mineral density; LS, least square; *n*, number of evaluable subjects; %CfB, percentage change from baseline. ^1^ LS means computed using weights for the strata as per representation in data.

**Table 3:** Statistical analysis of PD and PK denosumab parameters across treatment arms.

			Ratio of Geometric Means MB09/RP (%)	
**PD Parameter (Units)**	**Treatment**	**Geometric LS Means (*n*)**	**Estimate ^1^**	**90% CI**
MTP—AUEC_0–6 months_ (day·pg/mL)	MB09	11,700 (218)	99.91	(93.22, 107.08)
	RP	11,700 (228)		
TP—AUEC_0–6 months_ (day·pg/mL)	MB09-MB09	10,700 (184)	N/A	N/A
	RP-MB09	11,000 (101)	N/A	N/A
	RP-RP	9770 (93)	N/A	N/A
MTP—AUIC_0–6 months_ (day·%)	MB09	14,900 (218)	99.13	(96.76, 101.55)
	RP	15,100 (228)		
TP—AUIC_0–6 months_ (day·%)	MB09-MB09	15,500 (184)	N/A	N/A
	RP-MB09	15,500 (101)	N/A	N/A
	RP-RP	15,600 (93)	N/A	N/A
**PK Parameter (units)**	**Treatment**	**Geometric LS means (*n*)**	**Estimate**	**90% CI**
MTP—C_max_ (ng/mL)	MB09	5890 (269)	104.13	(99.66, 108.79)
	RP	5650 (273)		
TP—C_max_ (ng/mL)	MB09-MB09	5900 (228)	103.40 (MB09-MB09/RP-RP)	(96.28, 111.04)
	RP-MB09	5910 (125)	103.65 (RP-MB09/RP-RP)	(95.66, 112.31)
	RP-RP	5710 (111)		
MTP—AUC_0–6 months_ (day·ng/mL)	MB09	363,000 (256)	106.06	(100.82, 111.57)
	RP	342,000 (260)		
TP—AUC_0–6 months_ (day·ng/mL)	MB09-MB09	364,000 (214)	101.57 (MB09-MB09/RP-RP)	(93.68, 110.12)
	RP-MB09	356,000 (115)	99.48 (RP-MB09/RP-RP)	(90.80, 108.99)
	RP-RP	358,000 (104)		

Abbreviations: AUC_0–6 months_, area under the concentration-time curve over the first 6-month dosing interval; AUEC_0–6 months_, area under the effect curve from zero to 6 months; AUIC_0–6 months_, area under the inhibition curve from zero to 6 months; CI, confidence interval; C_max_, observed maximum serum concentration after study treatment administration; LS, least square; MTP, main treatment period; *n*, number of evaluable subjects; N/A, not applicable; PD, pharmacodynamic; PK, pharmacokinetic; RP, reference product; TP, transition period. ^1^ Results of AUEC_0–6 months_ are estimates of ratio of geometric means (MB09/RP) in sCTX AUEC_0–6 months_ in postmenopausal women with osteoporosis treated with subcutaneous denosumab injections every 6 months, assuming all women received their first denosumab dose without any errors in dosing and without receipt of any prohibited therapies or other osteoporosis medications up to 6 months after first dose.

**Table 4:** Summary of TEAEs during the MTP and Throughout the study (safety population).

	MTP		Throughout the Study		
Number of Subjects with	MB09 (*N* = 277) *n* (%)	Prolia (*N* = 278) *n* (%)	MB09-MB09 (*N* = 277) *n* (%)	Prolia-MB09 (*N* = 140) *n* (%)	Prolia-Prolia (*N* = 138) *n* (%)
Any TEAE	161 (58.1)	150 (54.0)	180 (65.0)	87 (62.1)	75 (54.3)
Any study treatment-related TEAEs	41 (14.8)	24 (8.6)	45 (16.2)	11 (7.9)	17 (12.3)
Any serious TEAEs	19 (6.9)	13 (4.7)	21 (7.6)	11 (7.9)	4 (2.9)
Any study treatment-related serious TEAEs	1 (0.4)	1 (0.4)	1 (0.4)	0	1 (0.7)
Any AESIs	80 (28.9)	75 (27.0)	96 (34.7)	47 (33.6)	43 (31.2)
Any serious AESIs	4 (1.4)	0	5 (1.8)	0	0
Any TEAEs leading to treatment discontinuation	4 (1.4)	0	4 (1.4)	0	0
Any TEAEs leading to death	0	0	1 (0.4)	0	0
Any deaths	0	0	1 (0.4)	0	0

Abbreviations: AESI, adverse event of special interest; MTP, main treatment period; *n*, number of evaluable subjects; TEAE, treatment-emergent adverse event.

## Data Availability

The datasets generated and/or analyzed during the current study are available from the corresponding author upon request.

## Supplementary Materials

The following supporting information can be downloaded at: https://www.mdpi.com/article/10.3390/pharmaceutics18030291/s1, Supplementary Methods: For PK analysis (denosumab serum concentrations)—MesoScale Discovery (MSD)-based ECL method, For PD analysis (serum carboxy-terminal cross-linking telopeptide of type I collagen [sCTX])—Enzyme-linked immunosorbent assay method (ELISA), Detection of anti-drug antibodies anti-denosumab and neutralizing antibodies in serum samples—MesoScale Discovery (MSD)-based ECL method; Supplementary Acknowledgments: List of investigators participating in the SIMBA study; Table S1: TEAEs reported in ≥2.0% of subjects in the MTP; Table S2: TEAEs reported in ≥1.0% of subjects in the TP.
