# Supplementary material for: Phase III Study to Confirm Clinical Similarity of MB09, a Denosumab Biosimilar, and Prolia® in Postmenopausal Women with Osteoporosis (SIMBA Study)
Source: Pharmaceutics. 2026 Feb 27;18(3):291. doi: 10.3390/pharmaceutics18030291 (PMC13028644; doi:10.3390/pharmaceutics18030291)
Supplement: Supplementary file 1 [file pharmaceutics-18-00291-s001.zip › Supplementary information-acknowledgements.pdf]

Supplementary information-acknowledgements

List of investigators participating in the SIMBA study:

Agita Medne, Health Center (Latvia); Airi Poder, Clinical Research Centre Ltd (Estonia); Andres Pille, East Tallinn Central Hospital (Estonia); Anna Sidorowicz- Bialynicka, AES - DRS - Synexus Polska Sp. z o.o. (Poland); Arija Lace, Outpatient Clinic Adoria (Latvia); Carlos Abud Mendoza, Hospital Central Dr Ignacio Morones Prieto (Mexico); Daniela Bichovska , AES - DRS - Medical Center Synexus Sofia EOOD (Bulgaria); Dorota Knychas, AES - DRS - Synexus Polska Sp. z o.o. (Poland); Edit Drescher, Vital Medical Center (Hungary); Edita Stokic, Clinical Centre of Vojvodina Hajduk Veljkova (Serbia); Elzbieta Kolodziejska, AES - DRS - Synexus Polska Sp. z o.o. (Poland); Emiliya Petkova, Diagnostic- Consultative Center Convex EOOD (Bulgaria); Eve-Kai Raussi, North Estonia Medical Centre Foundation (Estonia); Goran Radunovic, Institute of Rheumatology Belgrade – PPDS (Serbia); Ignacio Garcia-De la Torre, Centro de Estudios de Investigacion Basica Y Clinica SC (Mexico); István Szombati, QUALICLINIC Kft (Hungary); Ivan Goranov, Multiprofile Hospital for Active Treatment Plovdiv (Bulgaria); Ivaylo Lefterov, Medical Center Hera EOOD (Bulgaria); Ivo Valter, Center For Clinical and Basic Research (Estonia); Izabella Gladysz, Centrum Medyczne AMED (Poland); Janusz Jaworski, Centrum Medyczne Reuma Park NZOZ (Poland); Jose Gonzalez-Gonzalez, Hospital Universitario Dr. Jose Eleuterio González (Mexico); Kadri-Liina Vahula, KLV Arstikabinet Malmö 19 (Estonia); Katalin Guba, AES - DRS - Synexus Budapest - Magyarorszag Egeszsegugyi Szolgaltato Kft (Hungary); Katre Maasalu, Tartu University Hospital (Estonia); Krasimira Tsoneva, Medical Center Medconsult Pleven OOD (Bulgaria); Lali Kilasonia, Tbilisi Heart and Vascular Clinic Ltd (Georgia); Luba Lagvilava, Tbilisi Heart Center Ltd. Vazha-Pshavela (Georgia); Magdolna Nagy, Óbudai Egészségügyi Centrum Kft – Tiszaroff Lajos (Hungary); Mariela Geneva-Popova, Medical Center Artmed OOD (Bulgaria); Mariusz Korkosz, MCM Krakow - PRATIA – PPDS (Poland); Milan Petakov, University Clinical Center of Serbia – PPDS (Serbia); Milan Petronijevic, Military Medical Academy (Serbia); Nedyalka Staykova, Outpatient Clinic for Specialized Medical Help - Medical Center Kuchuk Paris OOD (Bulgaria); Nonna Nowak, ClinicMed Daniluk, Nowak Spólka Komandytowa (Poland); Paulina Ludziak, Globe Badania Kliniczne Spólka z o.o. (Poland); Pawel Miotla, Lubelskie Centrum Diagnostyczne (Poland); Péter Ditrói, AES - DRS - Synexus Debrecen - Magyarorszag Egeszsegugyi Szolgaltato Kft (Hungary); Péter Keszthelyi, Békés Vármegyei Központi Kórház Pándy Kálmán Tagkórház (Hungary); Piotr Ignaczak, Szpital Uniwersytecki Nr 2 im. Dr Jana Biziela w Bydgoszczy (Poland); Piotr Rozpondek, Krakowskie Centrum Medyczne (Poland); Raili Muller, MediTrials (Estonia); Renata Wysocka – Znojkiewicz, Centrum Medyczne Czestochowa - PRATIA – PPDS (Poland); Renate Helda, Sigulda Hospital (Latvia); Rodina Licheva, Specialized outpatient medical facility - Rheumatology Centre St. Irina EOOD (Bulgaria); Sandra Gintere, RSU Ambulance (Latvia); Sandra Miriam Carrillo Vazquez, Consultorio de Reumatologia Rio Bamba (Mexico); Tanya Aleksieva-Petkova, University Multiprofile Hospital for Active Treatment - Prof. Dr. Stoyan Kirkovich AD (Bulgaria); Tanya Tsvetanova, New Medical Center EOOD (Bulgaria); Tekla Szilassy, AES - DRS - Synexus Zalaegerszeg Magyarország Egészségügyi Kft (Hungary); Tomasz Dziewit, Centrum Medyczne Katowice - PRATIA – PPDS (Poland); Tzvetanka Petranova, Medical Center Excelsior OOD – PPDS 4 (Bulgaria); and Weronika Kleczynska- Szpakiewicz, Centrum Medyczne Linden (Poland).
