# Supplementary material for: Phase III Study to Confirm Clinical Similarity of MB09, a Denosumab Biosimilar, and Prolia® in Postmenopausal Women with Osteoporosis (SIMBA Study)
Source: Pharmaceutics. 2026 Feb 27;18(3):291. doi: 10.3390/pharmaceutics18030291 (PMC13028644; doi:10.3390/pharmaceutics18030291)
Supplement: Supplementary file 1 [file pharmaceutics-18-00291-s001.zip › Supplementary information-methods.pdf]

Supplementary information-methods

<u>For PK analysis (denosumab serum concentrations) - MesoScale Discovery (MSD)-based ECL method</u>

In this assay, electrochemiluminescent (ECL)-compatible plates were coated overnight with human receptor activator of nuclear factor kappa-B ligand (RANKL) protein. The sample denosumab (MB09) bound to the RANKL-coated wells, and sulfo tag-conjugated anti-denosumab antibodies were used to detect any bound antibodies and compared to a standard curve. This method assessed the biological similarity between MB09 and Xgeva® (EU- and US-sourced) and/or Prolia® (EU-sourced). The method was applicable to the quantitation of MB09 within a nominal range of 20.0 to 800 ng/mL, with anchor calibrators at 10.0 and 1000 ng/mL, and required a minimum 20.0 µL human serum aliquot. Samples were stored in polypropylene tubes and kept frozen at approximately -80 °C prior to analysis.

<u>For PD analysis (serum carboxy-terminal cross-linking telopeptide of type I collagen [sCTX]) - Enzyme-linked immunosorbent assay method (ELISA)</u>

CTx-1 was quantitatively measured from human serum using ELISA. In this assay, calibrators, quality controls, endogenous serum controls, assay blank, and unknown samples were added to a streptavidin coated plate, followed by the addition of an antibody solution. The antibody solution contained a biotinylated antibody and a horseradish peroxidase (HRP)-conjugated antibody. The biotinylated antibody and HRP-conjugated antibody form a complex between the CTx-1 that is found in the samples, calibrators, quality controls, and serum controls. The biotinylated antibody captures the complex to the plate by binding to the streptavidin coated plate. After incubation with the antibody solution, the plate was washed and a tetramethylbenzidine (TMB) solution was added to the plate. The TMB substrate solution reacted with the peroxidase, and in the presence of HRP, generated a colorimetric signal that was proportional to the amount of CTx-1 bound to the biotinylated antibody. The color development was stopped, and the colorimetric intensity (optical density) was measured at 450 nm. The minimum required dilution for this assay was 1:4 in antibody solution.

<u>Detection of anti-drug antibodies anti-denosumab and neutralizing antibodies in serum samples MesoScale Discovery (MSD)-based ECL method</u>

Anti-drug antibodies (ADAs) anti-denosumab and neutralizing antibodies (Nabs) were assessed using a validated MSD-ECL assay in a 3-Tiered approach. Cut point values (CPVs) were determined using upper prediction intervals. The CPVs were established in 1.14, 1.19, and 1.26 for screening, confirmation and titration tiers. Neutralizing antibodies were investigated only if a confirmed ADA positive result was obtained. The ADAs assay sensitivity was 2.99 ng/mL (low positive control of 12.0 ng/mL), with a drug tolerance of 2000 ng/mL in the presence of 500 µg/mL of denosumab. The Nabs assay sensitivity was 75.2 ng/mL (linear regression, low positive control of 140 ng/mL), with a drug tolerance of 1000 ng/mL nAb in the presence of 12.0 µg/mL of denosumab.
