# Supplementary material for: Phase III Study to Confirm Clinical Similarity of MB09, a Denosumab Biosimilar, and Prolia® in Postmenopausal Women with Osteoporosis (SIMBA Study)
Source: Pharmaceutics. 2026 Feb 27;18(3):291. doi: 10.3390/pharmaceutics18030291 (PMC13028644; doi:10.3390/pharmaceutics18030291)
Supplement: Supplementary file 1 [file pharmaceutics-18-00291-s001.zip › Supplementary Table S1.pdf]

Supplementary Table S1. TEAEs reported in ≥2.0% of subjects in the MTP (safety population)

PT	MB09 (N=277) n (%)	Prolia (N=278) n (%)	Total (N=555) n (%)
TEAEs reported in ≥2% subjects			
Upper respiratory tract infection	20 (7.2)	20 (7.2)	40 (7.2)
Arthralgia	15 (5.4)	15 (5.4)	30 (5.4)
COVID-19	14 (5.1)	15 (5.4)	29 (5.2)
Blood parathyroid hormone increased	11 (4.0)	4 (1.4)	15 (2.7)
Hypertension	10 (3.6)	6 (2.2)	16 (2.9)
Nasopharyngitis	10 (3.6)	23 (8.3)	33 (5.9)
Osteoarthritis	9 (3.2)	10 (3.6)	19 (3.4)
Urinary tract infection	9 (3.2)	9 (3.2)	18 (3.2)
Diarrhoea	7 (2.5)	4 (1.4)	11 (2.0)
Hypothyroidism	6 (2.2)	6 (2.2)	12 (2.2)
Back pain	6 (2.2)	7 (2.5)	13 (2.3)
Contusion	6 (2.2)	5 (1.8)	11 (2.0)

Abbreviations: COVID-19, coronavirus disease 2019; MTP, main treatment period; PT, preferred term; TEAEs, treatment-emergent adverse event.
