# Supplementary material for: Phase III Study to Confirm Clinical Similarity of MB09, a Denosumab Biosimilar, and Prolia® in Postmenopausal Women with Osteoporosis (SIMBA Study)
Source: Pharmaceutics. 2026 Feb 27;18(3):291. doi: 10.3390/pharmaceutics18030291 (PMC13028644; doi:10.3390/pharmaceutics18030291)
Supplement: Supplementary file 1 [file pharmaceutics-18-00291-s001.zip › Supplementary Table S2.pdf]

Supplementary Table S2. TEAEs reported in ≥1.0% of subjects in the TP (safety population)

PT	Prolia-MB09 (N=130) n (%)	Prolia-Prolia (N=123) n (%)	Total (N=253) n (%)
TEAEs reported in ≥1% subjects			
Upper respiratory tract infection	5 (3.8)	2 (1.6)	7 (2.8)
COVID-19	3 (2.3)	3 (2.4)	6 (2.4)
Nasopharyngitis	2 (1.5)	2 (1.6)	4 (1.6)
Bronchitis	1 (0.8)	2 (1.6)	3 (1.2)
Urinary tract infection	1 (0.8)	2 (1.6)	3 (1.2)
Hypertension	1 (0.8)	3 (2.4)	4 (1.6)
Obesity	1 (0.8)	2 (1.6)	3 (1.2)

Abbreviations: COVID-19, coronavirus disease 2019; PT, preferred term; TEAEs, treatment-emergent adverse event; TP, transition period
